# Discordant Dose-Dependent Metabolic Effects of Eicosapentanoic Acid in Diet-Induced Obese Mice

**DOI:** 10.3390/nu12051342

**Published:** 2020-05-08

**Authors:** Mandana Pahlavani, Latha Ramalingam, Emily K. Miller, Hanna Davis, Shane Scoggin, Naima Moustaid-Moussa

**Affiliations:** Department of Nutritional Sciences, and Obesity Research Institute, Texas Tech University, Lubbock, TX 79409, USA; mandana.pahlavani@ttu.edu (M.P.); latha.ramalingam@ttu.edu (L.R.); emily.k.miller@ttu.edu (E.K.M.); hanna.davis@ttu.edu (H.D.); shane.scoggin@ttu.edu (S.S.)

**Keywords:** eicosapentaenoic acid, dose-response, glucose homeostasis, mice, obesity, omega 3 fatty acids

## Abstract

Obesity is a widespread epidemic that increases the risk for several metabolic diseases. Despite several beneficial health effects of eicosapentaenoic acid (C20:5n-3, EPA), previous studies have used very high doses of EPA. In this study, dose-dependent effects of EPA on metabolic outcomes were determined in diet-induced obese mice. We used B6 male mice, fed high-fat diet (HF, 45% kcal fat) or HF diet supplemented with 9, 18, and 36 g/kg of EPA-enriched fish oil for 14 weeks. We conducted metabolic phenotyping during the feeding period, and harvested tissues and blood at termination. Only mice fed 36 g/kg of EPA significantly (*p* < 0.05) lowered body weight, fat content and epididymal fat pad weight, compared to HF. Both 18 and 36 g/kg doses of EPA significantly increased glucose clearance and insulin sensitivity, compared to HF or 9 g/kg of EPA. Locomotor activity was significantly increased with both 18 and 36 g/kg doses of EPA. Interestingly, all doses of EPA compared to HF, significantly increased energy expenditure and oxygen consumption and significantly reduced serum insulin, leptin, and triglycerides levels. These results demonstrate weight- and adiposity-independent metabolic benefits of EPA, at doses comparable to those currently used to treat hypertriglyceridemia.

## 1. Introduction

The growing prevalence of obesity across the world increases risk of metabolic and cardiovascular diseases, and obesity is now recognized as a disease of epidemic proportions [1,2]. It increases risk of co-morbidities including type II diabetes, insulin resistance, heart diseases and some cancers [3]. In obesity, white adipose tissue (WAT) becomes enlarged and inflamed resulting in infiltration of macrophages [4]. This, in turn, increases levels of circulating inflammatory markers, such as monocyte chemoattractant protein 1 (MCP-1), tumor necrosis factor (TNFα), and interleukin 6 (IL-6), which increase the macrophage infiltration leading to a vicious cycle [5]. 

Dietary energy restriction and increased physical activity are recommended lifestyle strategies for reducing obesity. These are efficient, but compliance is an issue [6]. An alternative approach to obesity prevention is intake of bioactive compounds with anti-obesity and anti-inflammatory properties [7]. We are specifically interested in long-chain ω3 polyunsaturated fatty acids (PUFA) that offer numerous beneficial effects, which include lowering inflammation, hypertriglyceridemia and conferring cardiovascular benefits [8]. While weight reduction with fish oil has been reported mostly in animal models, other weight-independent metabolic benefits have been documented in both animals and humans [9,10]. 

Marine-based fish and fish oil are the most popular sources of PUFA: namely, eicosapentaenoic acid (C20:5n-3, EPA), docosapentaenoic acid (C22:5n-3, DPA), and docosahexaenoic acid (C22:6n-3, DHA) [11,12,13]. They are essential fatty acids, as humans and animals cannot synthesize them [14]. Interventions using EPA, DPA, or DHA are important as conversion from α-linolenic acid (ALA) to EPA is limited [15], since only about 0.2–8% of ALA is converted to EPA and only 0–4% of ALA is converted to DHA [16,17]. Downstream lipid mediators of EPA and DHA include resolvins, protectins, and maresins, which also exert inflammation resolving effects [18,19,20,21,22].

Previous work from our lab has demonstrated that feeding mice a high-fat (HF) diet enriched with EPA significantly reduced body weight, fat mass, and inflammation, and increased insulin sensitivity compared to HF-fed mice [22]. These effects are, in part, due to reduced WAT inflammation and lipogenesis, and increased brown adipose tissue thermogenic markers [22,23,24,25]. However, in our previous study, the amount of EPA (36 g/kg of diet) used for mice diet was relatively high. This dose represented 6.7% of total mouse energy intake for diet-induced obese mice fed 45%kcal fat dies. For human being, with an estimated 35% kcal fat in the diet, this amount of EPA is estimated as 5.22% of total energy intake; additional mouse to human dose translations are included in the discussion section. Some metabolic effects of fish oil have been addressed in other studies [26,27]. However, considering availability of both fish oil (DHA/EPA combo) as well as EPA only as anti-hypertriglyceridemic agents, it is very important to further dissect effects of EPA (less studied alone compared to fish oil). Moreover, it is difficult to compare between individual studies, which used different doses of EPA due to differences in source and composition of fish oil or EPA products used, amount of fat (low or high) or percent of high fat (45% or 60%) [28,29]. Here, we conducted a dose-dependent study in a diet-induced obese mouse model to understand metabolic effects of various doses of EPA within ranges of fish oil supplementation used in human studies [30,31] and including amounts consistent with therapeutic doses of fish oil EPA or EPA+DHA) used for hypertriglyceridemia [32]. Specifically, we compared doses of EPA ranging from 9 g EPA/kg of mouse diet (EPA9; 1.67% kcal fat of mouse energy intake), to a dose of 18g/kg of diet (EPA18; 3.35% kcal fat), and to previously used high dose of 36 g/kg mouse diet (EPA36, 6.7% kcal fat) [22]. Various methods to translate these doses into human intakes are presented in the discussion section. Our primary goal was to determine the lowest effective dose of EPA that exerts comparable metabolic benefits to the high dose of EPA36, We found that EPA18 is as effective as EPA 36 for major physiological effects of EPA in diet-induced obese mice, and both EPA9 and EPA18 exerted several metabolic benefits that are independent of body weight and adiposity. Moreover, our findings indicate that doses comparable to those used in humans to treat hypertriglyceridemia [32] confer added metabolic benefits in obesity.

## 2. Materials and Methods 

### 2.1. Diets and Study Design

C57BL/6J male mice, aged 5–6 weeks, were purchased from the Jackson Laboratory (Bar Harbor, ME, USA) and were randomized by weight into different groups (15 mice per group). Mice were either fed a high-fat (HF) diet (45%, 20%, and 35% of energy from fat, protein, and carbohydrate, respectively) or an HF diet supplemented with 9, 18 and 36g/kg (EPA9, EPA18 and EPA36, respectively) of AlaskOmega EPA-enriched fish oil (800 mg/g), (provided by Organic Technologies, Coshocton, OH, USA) for up to 14 weeks. Detailed diet composition is provided in Supplementary Table S1. Animals were housed in individual ventilated cages (IVC) at 22 °C with a 12-h light/dark cycle and free access to food and water. Food was replaced twice each week to avoid rancidity, and food intake was measured by assessing the amount of food consumed. Fatty acid composition as well as oxidation of fish oil and diet were measured to ensure integrity. Glucose tolerance test (GTT), insulin tolerance test (ITT), body composition, and energy expenditure were performed during the feeding period as shown in Table 1. The dietary intervention was for 14 weeks. However, during the last two weeks, we measured energy expenditure using metabolic cages and did not record body weight. At the end of the 14 weeks, all animals were euthanized following a 5-h fast using CO_2_ inhalation method. The epididymal fat pad and other tissues were collected, snap-frozen in liquid nitrogen, and stored at −80 °C until further analyses. All animal protocols were approved by the institutional animal care and use committee (IACUC) of Texas Tech University. The Protocol number is 19034-04 and was approved on 1 April 2019.

### 2.2. Triglyceride Assay

Triglycerides colorimetric assay kit (Cayman, Ann Arbor, MI, USA) was used to determine the triglyceride content in serum, according to manufacturer’s guidelines.

### 2.3. Red Blood Cells Fatty Acid Composition

Gas chromatography/mass spectrometry method and direct fatty acid methyl ester (FAME) synthesis were performed as previously described to determine changes in fatty acid composition [33,34].

### 2.4. Glucose and Insulin Tolerance Tests

Mice ten weeks of age were fasted at 8 am for 5 h for GTT, previously established as an appropriate duration of fasting in rodents for insulin resistance studies [35]. Blood glucose levels were measured at 0, 15, 30, 60, 90, and 120 min after intraperitoneal (i.p.) injection of glucose (2 g kg^−1^ body weight), using OneTouch Ultra GlucoMeter (AlphaTrak, North Chicago, IL, USA). After 12 weeks of dietary intervention, ITT was conducted, following 5 h fasting. Mice were injected with 1U kg^−1^ insulin (Humulin, Indianapolis, IN, USA). Blood glucose levels were then measured at 0, 15, 30, 45, 60, and 90 min. The area under the curve was calculated using the trapezoidal method with values normalized to the lowest basal glucose value. 

### 2.5. Body Composition

Body fat and lean mass percentage determination was performed using Echo-MRI instrument (EchoMRI LLC, Houston, TX, USA) during week 11 of dietary intervention.

### 2.6. Metabolic Cages and Energetics

Thirteen weeks old mice were acclimated in metabolic chambers (TSE Systems, Hamburg, Germany) for 2 days. Mice were continuously recorded for 4 days to monitor 24 h respiratory parameters under a 12 h light–dark cycle with the following measurements taken every 50 min: water intake, food intake, ambulatory activity (in X and Z axes), and gas exchange (O_2_ and CO_2_). Heat (H) and locomotor activity (Dist H and K) were calculated based on the actual weight of the animal. Home-cage locomotor activity was determined using an ActiMot infrared light beam system integrated in the calorimetry system. Room temperature was controlled at 23 ºC in an isolated environment.

### 2.7. Plasma Insulin and Adipokine Measurements

Insulin and leptin levels were measured using a commercially available microsphere-based multiplexing system (Luminex xMAP) as described previously [22].

### 2.8. Statistical Analysis

All data are presented as mean ± SEM. For multiple comparisons, one-way ANOVA followed by Tukey’s post hoc test (non-parametric; *p <* 0.05) was performed,). All statistical tests were performed using GraphPad Prism software.

## 3. Results

### 3.1. Effects of EPA on Food Intake, Body Weight, Fat Pad Weight, and Body Fat Percentage 

**Figure 1:**
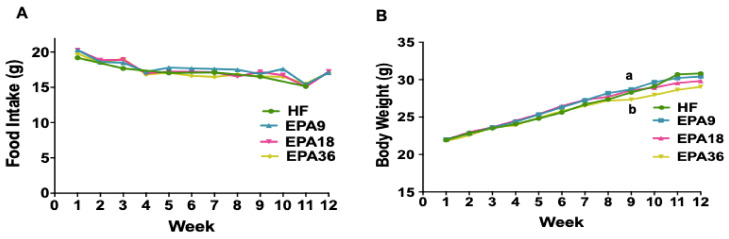
Effect of EPA on food intake and body weight among four groups of mice (HF, EPA: 9,18, and 36 g/kg. (**A**) No significant differences in food intake (average per week per mouse) among four group of diets, (**B**) EPA36 significantly reduced body weight after the ninth week compared to HF. Data are expressed as mean ± SEM. Groups represented with same letter indicate no difference. *p* < 0.05; *n* = 15 mice per group. EPA, eicosapentaenoic acid; HF, high fat.

We have previously reported that mice fed a high-fat diet supplemented with EPA36 (36 g EPA/kg diet) displayed an improved overall metabolic profile, independent of obesity [22]. Here, we tested whether 9 and 18 g/kg of EPA showed similar effects as 36 g/kg EPA compared to HF. Food intake was measured per mice weekly, and it was comparable between HF, EPA9, EPA18, and EPA36 throughout the feeding period (Figure 1A). However, EPA36 significantly decreased body weight (BW) compared to HF and EPA9 groups after the 9th week until the 12th week of dietary intervention (Figure 1B).

**Figure 2:**
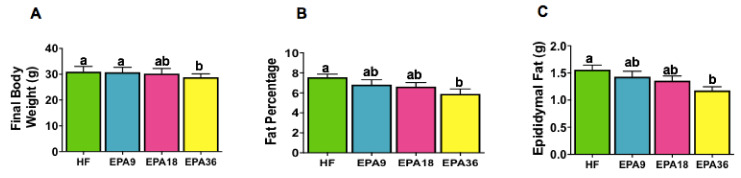
Dose-dependent effects of EPA on final body weight, fat percentage, and epididymal fat pad weight in mice. (**A**) EPA36 significantly reduced body weight compared to HF, (**B**) EPA36 significantly reduced fat percentage compared to HF. (**C**) EPA36 significantly reduced epididymal fat pad weight compared to HF. Data are expressed as mean ± SEM. Groups represented with same letter indicate no difference. *p* < 0.05; *n* = 15 mice per group. EPA, eicosapentaenoic acid; HF, high fat.

When comparing final body weights at euthanasia (14th week of dietary intervention) between the four groups, only EPA36 showed a significant decrease in final body weight compared to EPA9 and HF (Figure 2A). No significant differences in final body weights were observed between EPA18 and EPA36. Mice fed EPA36 also had a significant reduction in epididymal fat pad and fat percentage compared to HF (Figure 2B,C). No significant differences in body fat percentage or epididymal fat pad weight were observed among EPA9, EPA18 or HF. Taken together, these results suggest that mice fed EPA36 but not lower doses had reduced weight gain and adiposity compared to the HF group.

### 3.2. Effects of EPA on Glucose Homeostasis 

**Figure 3:**
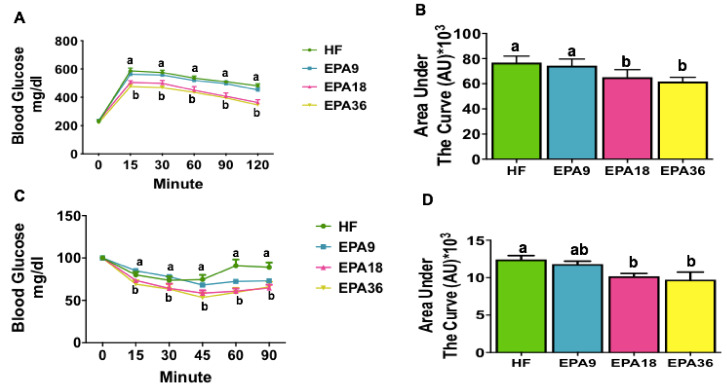
Dose-dependent effects of EPA on glucose homeostasis in mice. (**A**) EPA18 and 36 significantly increased glucose clearance in GTT compared to HF and EPA 9, (**B**) Area under the curve of GTT. (**C**) EPA18 and 36 significantly improved insulin sensitivity in ITT compared to HF and EPA 9, (**D**) Area under the cure of ITT. Data are expressed as mean± SEM. Groups represented with same letter indicate no difference. *p* < 0.05; *n* = 15 mice per group. EPA, eicosapentaenoic acid; GTT, glucose tolerance test; HF, high fat, ITT, insulin tolerance test.

In order to determine the effect of different doses of EPA on glucose homeostasis, we performed GTT and ITT. Both EPA18 and EPA36 fed mice had significantly increased glucose clearance compared to HF and EPA9 at all time points (Figure 3A). This was consistent with the area under the curve data as shown in Figure 3B. The results from ITT showed that when compared to HF, only EPA36 significantly reduced glycemia by 45 min. However, at 60 and 90 min, both EPA18 and EPA36 significantly reduced glycemia compared to HF and EPA9 (Figure 3C,D.)

### 3.3. Effects of EPA on Obesity-Associated Metabolic Hormones and Triglycerides

**Figure 4:**
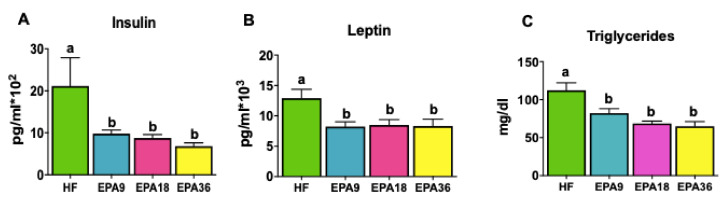
Dose-dependent effects of EPA on serum insulin, leptin, and triglycerides levels in mice**.** (**A**) EPA9, 18, and 36 significantly decreased the insulin level compared to HF, (**B**) EPA9, 18, and 36 significantly decreased the leptin levels compared to HF. (**C**) EPA9, 18, and 36 significantly reduced triglycerides levels in serum compared to HF. Data are expressed as mean ± SEM. Groups represented with same letter indicate no difference. *p* < 0.05; *n* = 8– 15 mice per group. EPA, eicosapentaenoic acid; HF, high fat.

Serum insulin levels were measured given the importance of this hormone in glucose and energy metabolism [36] and changes observed in GTT and ITT. Interestingly, EPA significantly reduced fasting insulin levels for all three doses (*p* < 0.05) compared to HF (Figure 4A). We also measured levels of leptin as an adipose-derived peptide hormone [37]. Consistent with changes in insulin, EPA significantly reduced fasting leptin levels at all three doses (*p* < 0.05) compared to HF (Figure 4B). We also examined the effects of EPA on serum triglycerides and demonstrated that all EPA-treated groups had significantly lower triglycerides levels (*p* < 0.05) compared to HF (Figure 4C).

### 3.4. Effect of EPA on Energy Homeostasis

**Figure 5:**
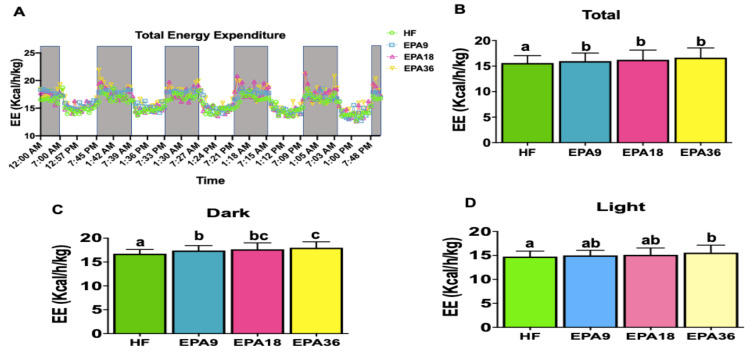
Dose-dependent effects of EPA on energy expenditure in mice. (**A**,**B**) EPA9, 18, and 36 significantly increased total energy expenditure compared to HF, (**C**) EPA9, 18, and 36 significantly increased energy expenditure compared to HF in dark, (**D**) EPA36 significantly increased energy expenditure compared to HF in light. Data are expressed as mean ± SEM. Groups represented with same letter indicate no difference. *p* < 0.05; *n* = 4 mice per group. EE, energy expenditure; EPA, eicosapentaenoic acid; HF, high fat.

**Figure 6:**
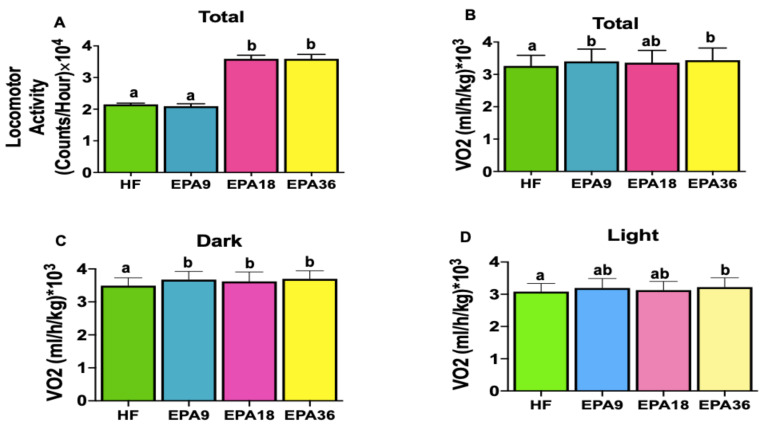
Dose-dependent effects of EPA on locomotor activity and oxygen consumption in mice. (**A**) EPA18 and 36 significantly increased total locomotor activity compared to HF and EPA9, (**B**) EPA9 and 36 significantly increased total oxygen consumption compared to HF, (**C**) EPA9, 18, and 36 significantly increased oxygen consumption compared to HF in dark, (**D**) EPA36 significantly increased oxygen consumption compared to HF in light. Data are expressed as mean ± SEM. Groups represented with same letter indicate no difference. *p* < 0.05; *n* = 4 mice per group. EPA, eicosapentaenoic acid; HF, high fat.

To investigate the effects of EPA on energy homeostasis we performed indirect calorimetry measurements. Total energy expenditure at all EPA doses was significantly increased compared with HF group (Figure 5A,B). During dark cycle, all EPA doses showed significantly higher energy expenditure than HF while, only EPA36 demonstrated significant increases in energy expenditure compared to HF during light cycle (Figure 5C,D). However, no significant changes in respiratory exchange ratio (RER) were observed across the four groups. Cumulative (Supplementary Figure S1) and total locomotor activity demonstrated significant increases in both EPA18 and 36 compared to EPA9 and HF over the four days (Figure 6A). Total oxygen consumption in EPA9 and 36 groups was also significantly increased compared with HF group (Figure 6B). During dark cycle, all EPA doses showed significantly higher oxygen consumption compared to HF, while only EPA36 demonstrated significant increases in oxygen consumption compared to HF during the light cycle (Figure 6C,D).

### 3.5. Fatty Acid Analysis in Mice Serum with Different Diets

Fatty acid analysis of RBC showed significant increases in EPA levels across all doses of EPA (9, 18, and 36) compared to HF as shown in Table 2. 

## 4. Discussion

Several studies have shown that n-3 PUFA exert potent biological effects [22,26,27,38] in metabolic diseases in mouse models and in some clinical studies [8,9,10,22,29,39]. Studies showed that dietary n-3 DPA, which is a source of EPA and DHA, is implicated in the pro-resolution of inflammation and decreased lipid parameters [13,40,41,42]. We previously reported that feeding mice a HF diet enriched with 36 g/kg EPA (EPA36) significantly reduced body weight, fat mass, glucose intolerance, inflammation, insulin resistance, and increased DPA level [26] compared to HF-fed mice [22,26]. Several of these protective effects were independent of obesity, such as reduced liver steatosis and inflammation [22,26,43].

Consistent with these findings, we showed here that EPA36 significantly reduced BW, epididymal fat pad weight, fat percentage, glucose clearance, insulin resistance, serum triglycerides, leptin, and insulin levels, compared to the HF diet. However, diet-induced obesity effects observed in the current study are less pronounced that those previously reported [22], possibly due to: (1) different environments as former study was performed at the University of TN, and current study at Texas Tech university); (2) source of EPA (previously we used pure EPA ethyl ester, and current study used fish oil at 800mg EPA/g from different sources; and (3) While mice used in both studies are inbred B6 mice, different generations and litters may exhibit different dietary responses. 

We also demonstrated that EPA36 significantly increased energy expenditure, locomotor activity, and oxygen consumption, which may explain the body weight reduction in this group, compared to HF. While these studies contribute significant knowledge about metabolic effects of fish oil, a major limitation was the relatively high dose of EPA used. The objective of this work was to specifically determine the lowest effective dose of EPA on various metabolic parameters related to obesity and energy/glucose homeostasis compared to EPA36. Our data are summarized in Table 3.

Rossmeisl et al. reported reduced weight gain and insulin resistance in B6 mice fed HF diet supplemented with 5 g/kg DHA/EPA for 7 weeks compared to HF (35% diet) [44]. Another study showed that B6 mice fed HF diet (60% of energy), supplemented with fish oil (7.5 % of fat replaced) for 14 weeks had no effect on BW compared to HF, but increased levels of fatty acid oxidation genes in skeletal muscle [45]. Consistent with this study, we did not observe significant reduction in BW with EPA18; however, we observed significant improvements in glucose tolerance and insulin sensitivity, as assessed by GTT and ITT, respectively. The Dai et al. study reported an increase in O_2_ consumption, CO_2_ production, and heat production in rats diet supplemented with fish oil (6% of weight) compared to soy oil diet (6% of weight) for 13 weeks [46]. Consistent with this study, we demonstrated significant increases in energy expenditure, oxygen consumption, and locomotor activity with EPA18 (7.5% of weight) compared to HF, although this dose did not reduce body weight.

Cussons et al. investigated the effects of 4 g/day fish oil (27% EPA and 56% DHA) in polycystic ovarian syndrome (PCOS) women over 8 weeks [47]. They observed significant decreases in waist circumference (WC), body weight, body mass index (BMI), fat mass, and fasting glucose in the fish oil group compared to control [47]. Another study used 4 g/day of n-3 PUFA (46% EPA and 38% DHA) in 27 individuals with obesity for 16 weeks [48] and observed significant reductions in BW, BMI, WC, and homeostatic model assessment-insulin resistance (HOMA-IR) in fish oil group compared to a control group (hypocaloric diet) [48]. In contrast to these studies, no significant differences were reported in BW, BMI, and WC between fish oil and control groups, but improvement in serum glucose and insulin were reported in PCOS subjects using 4 g/day fish oil (720mg EPA and 480mg DHA, 4 capsules each day) for 8 weeks [49,50]. One possible explanation for this discrepancy in these two studies is the difference in amounts and composition of the fish oil fatty acids. In addition, these clinical studies used fish oil enriched with both EPA and DHA, compared to our study, where we used fish oil enriched primarily in EPA (80%). Furthermore, different intervention times and genetic variability in mice and humans could be possible explanations for these discrepancies.

Islam et al. reported that locomotor activity did not change in B6 mice supplemented with 2% menhaden oil compared to a low fat diet for 12 weeks [51]. In line with our study, EPA (1% w/w) reduced the leptin level in B6 male mice, fed for 16 weeks [28]. Crochemore et al. showed that 2.5 g/d PUFA (574.5 mg EPA and 352.5 mg DHA) had no effects on BW, BMI, or glucose level on 45 post-menopausal subjects for 4 weeks [52]. In accordance with this study, no significant differences in BW were observed among 128 subjects with obesity, who were supplemented with omega-3 (3 g/day, EPA/DHA 5:1) in comparison with a placebo for 24 weeks [53]. There were no significant differences in resting metabolic rate and fatty acid oxidation in human subjects using 3 g/day fish oil (2 g/day EPA and 1 g/day DHA), for 12 weeks [54]. In another clinical study, no significant effects of EPA (1.8-2.7 g/day) on steatosis, inflammation, and insulin resistance were observed [55]. In line with these clinical data, we did not observe significant changes in glucose tolerance or insulin sensitivity with 9 g/kg EPA compared to HF.

While leptin levels are closely matched to body adiposity, increased energy expenditure and reduced triglycerides in serum [56] with EPA9 and 18 may explain the reduced leptin levels without significantly reducing BW and adiposity compared to HF, as the negative energy balance produces a fall in the leptin level, which is more rapid than change in adiposity [57]. Additionally, EPA9 significantly decreased fasting insulin, leptin, and triglyceride levels and significantly increased energy expenditure and oxygen consumption compared to HF which is comparable to healthy obese subjects criteria [58]. These findings confirm the beneficial metabolic effects of EPA even at a lower dose (9g/kg in mice diet and 2.5 g/day in human diet) which is generally used as a supplement for healthy humans. Thus, our dose response study may help resolve the previous conflicting findings of low dose fish oil.

The groups EPA18 and EPA9 had lower amounts of EPA than EPA36. Hence, they may need more time to show significant decreases in BW, fat percentage, and epidydimal fat pad weight compared to HF despite showing increased energy expenditure.

The HF diet (45% of energy) used for this study is comparable to diets used in other animal studies that mimic western diets, with 40%–42% kcal fat [59,60]. These recommended diets are still higher than the Dietary Reference Intakes (DRI) for fat that is estimated at 20%–35% kcal fat [61]. The amount of saturated fatty acids (lard) in EPA36, 18, and 9 groups were decreased by 22%, 11%, and 5.5% compared to HF group respectively and replaced with EPA. Hence, it needs to be noted that metabolic effects of decreasing the amount of lard could also contribute to the observed effects while the amount of saturated fatty acids (SFA) in the red blood cells did not change across four groups in fatty acid analyses.

We showed that EPA18 is as effective as EPA 36 for major physiological effects of EPA reported here. Several methods estimate human equivalent doses based on mouse diets. In our study and previous ones [22,26], we estimated this dose based on caloric intake of fat as follows: The 18 g EPA /kg mouse diet represents 3.35% of total energy for mice fed 45% kcal fat diets. For humans, if we considered the upper DRI intakes of 35% kcal fat diets for human intake, the amount of EPA is 2.6% kcal EPA (total energy), or 52 kcal based on a 2000 kcal daily energy intake, or ~5.2 g /day of EPA. However, other methods have been recommended in recent years, for translating mouse doses to humans. One of these methods (allomeric scaling) is based on body surface and body weights for mice (or other animal models) compared to humans [62,63]. If we consider this method, our dose of 18 g EPA/kg diet (for a 30g mouse and 2.5g food intake per day) is estimated to be 7 g /day of EPA for a 60 kg human body weight.

We normalized energy expenditure to lean body mass to eliminate the effect of different body size as a common approach [64,65]; however, a study showed that this is not an appropriate approach because the regression line between body mass and metabolic rate does not have a zero intercept, and is therefore not linear [66]. Instead, using analysis of covariance (ANCOVA) was recommended for normalizing metabolic rate to body mass. This approach allows the combination of discrete (for example, genotype) and continuous (for example, body mass) traits as a calculation of energy expenditure [66]. In our case, body weights were comparable among most groups, and only body weights of mice fed EPA36 were significantly lower than the other 3 dietary groups.

The changes in locomotor activity did not significantly impact daily energy expenditure in this study. One possible explanation is related to the standard laboratory housing conditions of 20–24 °C with ad libitum access to food. At temperatures below thermoneutrality, activity has being shown to have an independent effect on total energy expenditure; in other words, mice that moved more than others did not necessarily expend more energy [67]. The lack of relationship between energy expenditure from activity and total energy expenditure may be due to other thermogenic processes, such as decreased thermogenesis, which might mask the effect of activity at below thermoneutrality conditions [67].

This study’s major strength is that it is the first to determine dose-dependent effects of EPA on markers of body weight, adiposity, glucose homeostasis, and energy balance. However, our study has some limitations which include not using fish oil with both EPA and DHA; this is worth investigating and comparing to whole fish oil or fish consumption. Since EPA is prescribed as a hypotriglyceridemic agent, our findings are still relevant. In addition, limitations in techniques which we utilized to determine small differences in energy and adiposity should be considered for the contradiction between increasing locomotor activity with no changes in energy expenditure in the EPA9 group. We used male mice as we previously showed that in B6 male mice, obesity-associated metabolic dysfunctions such as glucose intolerance, insulin resistance, and adiposity are more pronounced in males. However, performing future studies in females is warranted both in animal and human studies.

## 5. Conclusions

In previous studies, EPA 36 g/kg was effective in reducing obesity and related inflammation and insulin resistance [22,26]. However, this EPA dose exceeds and is more than double the recommended level of EPA for hypertriglyceridemic patients (4 g/d). No studies have been conducted on the dose-dependent effects of EPA in diet-induced obese mice. Here, we tested 9, 18 and 36 EPA (g/kg) diets. We demonstrated that EPA18 is as effective as EPA 36 for major physiological effects of EPA such as reducing glycemia, triglycerides, insulin, and leptin levels, and increasing insulin sensitivity, energy expenditure, locomotor activity, and oxygen consumption. This dose of EPA (18 g/kg mouse diet, approximately 5 g/d in human diet) is more closely consistent with the currently recommended amounts (4 g/day) of omega 3 fatty acids in patients with hypertriglyceridemia as a therapeutic intervention [32,68,69]. As EPA18 did not significantly change body weight or adiposity, these results indicate that obesity-independent effects of EPA may improve metabolic health, with some beneficial effects achieved at lower doses than at doses used for hypertriglyceridemia.

## Figures and Tables

**Table 1:** Study timeline.

Procedure	Start of Diet (Mice Age 5–6 weeks)	GTT	Body Composition	ITT	Metabolic Cage	Euthanasia
Time (weeks)	0	10	11	12	13	14

**Table 2:** Fatty acid composition of RBC in C57BL/6J mice in the HF, EPA9, 18, and 36 groups (mol%).

	HF	EPA9	EPA18	EPA36	*p*-Value
Saturated Fatty Acids	40.94 ± 3.7	46.22 ± 4.1	46.76 ± 4.2	50.17 ± 4.6	NS
C15:0 pentadecanoic acid	0.22 ± 0.22	0.53 ± 0.27	0.70 ± 0.05	0.48 ± 0.24	NS
C16:0 palmitic acid	25.52 ± 0.77 ^a^	28.76 ± 0.37 ^b^	29.84 ± 0.61 ^b^	32.29 ± 0.64^bc^	<0.01
C18:0 octadecanoic acid	15.20 ± 0.16 ^a^	16.93 ± 0.18 ^ab^	16.22 ± 0.05 ^ab^	17.40 ± 0.27 ^b^	<0.02
Monosaturated Fatty Acids	26.36 ± 2.3	19.12 ± 2	19.61 ± 2	17.72 ± 2.3	NS
C17:1 Heptadecenoic acid	0.160 ± 0.27	0.35 ± 0.70	0.45 ± 0.19	0.00	NS
C18:1, trans Elaidic acid	12.29 ± 1.56 ^a^	5.16 ± 0.91^b^	5.60 ± 1.45 ^b^	2.71 ± 0.43 ^b^	<0.01
C18:1, cis oleic acid	13.91 ± 1.72	13.61 ± 0.19	13.56 ± 0.35	15.01 ± 0.27	NS
PUFA	25.51 ± 1.35	32.4 ± 0.52	30.36 ± 0.82	28.9 ± 0.92	NS
ω6 Fatty Acids	22.07 ± 1.9	22.56 ± 1.4	18.04 ± 1.2	14.19 ± 1	NS
C18:2, trans linolelaidic acid	0.80 ± 0.40	1.14 ± 0.01	1.20 ± 0.02	1.11 ± 0.02	NS
C18:2, cis linoleic acid	10.32 ± 0.81 ^ab^	10.76 ± 0.16 ^a^	9.51 ± 0.11 ^ab^	8.83 ± 0.11 ^b^	<0.04
C20:3 eicosatrienoic acid	0.00 ^a^	0.59 ± 0.02 ^b^	0.16 ± 0.16 ^a^	0.00^a^	<0.003
C20:4 arachidonic acid	10.95 ± 5.16	10.07 ± 0.11	7.17 ± 0.19	4.25 ± 0.09	NS
ω3 Fatty Acids	3.44 ± 0.8	9.84 ± 0.1	12.32 ± 0.5	14.71 ± 1.4	NS
C20:5 eicosapentaenoic acid (EPA)	0.00 ^a^	4.66 ± 0.09 ^b^	7.31 ± 0.37 ^c^	10.58 ± 0.16 ^d^	<0.0001
C22:6 docosahexaenoic acid (DHA)	3.44 ± 0.17 ^a^	5.18 ± 0.05 ^b^	5.01 ± 0.10 ^b^	4.13 ± 0.06 ^c^	<0.0001

Data are expressed as mean ± SEM. Groups represented with same superscript letter indicate no difference. *p* < 0.05; *n* = 3 mice per group. EPA, eicosapentaenoic acid; HF, high fat, NS, non-significant.

**Table 3:** Summary of dose-dependent effects of EPA on various metabolic outcomes in mice (compared to HF control). (up): significantly increased (*p* < 0.05) compared to HF; (down): significantly decreased (*p* < 0.05) compared to HF (NS): no significant difference from HF. EPA, eicosapentaenoic acid; HF, high fat; NS, no significant.

	Body Weight	Fat Percentage	Glycemia (GTT)	Triglycerides	Fasting Insulin	Fasting Leptin	Energy Expenditure	Oxygen Consumption	Locomotor Activity
EPA9	NS	NS	NS	Down	Down	Down	Up	Up	NS
EPA18	NS	NS	Down	Down	Down	Down	Up	NS	Up
EPA36	Down	Down	Down	Down	Down	Down	Up	Up	Up

## Supplementary Materials

The following are available online at https://www.mdpi.com/2072-6643/12/5/1342/s1, Figure S1: Dose-dependent effects of EPA on locomotor activity in mice. EPA18 and 36 significantly increased cumulative locomotor activity compared to HF and EPA9. Table S1: Diet compositions. EPA, eicosapentaenoic acid; HF, high fat.
