# Supplementary figures and images for: Discordant Dose-Dependent Metabolic Effects of Eicosapentanoic Acid in Diet-Induced Obese Mice
Source: Nutrients. 2020 May 8;12(5):1342. doi: 10.3390/nu12051342 (PMC7284763; doi:10.3390/nu12051342)

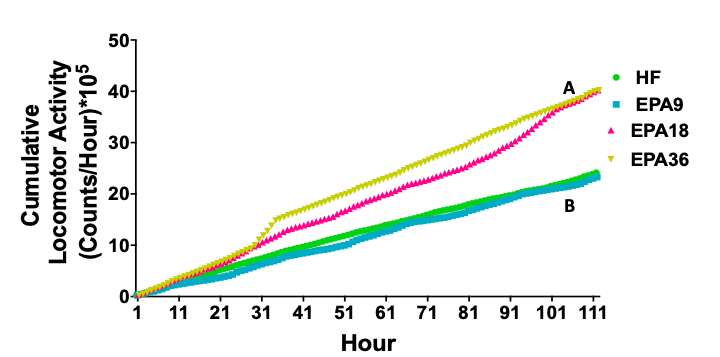

Supplement: Supplementary file 1 [file nutrients-12-01342-s001.zip › Supplementary Figure 1.tiff]

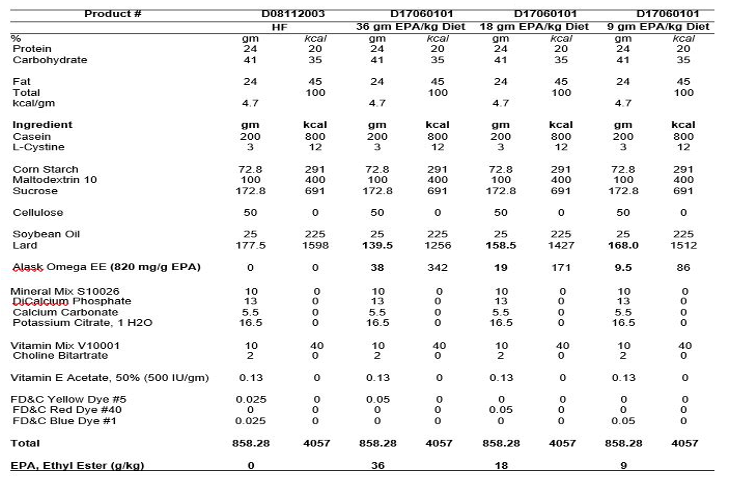

Supplement: Supplementary file 1 [file nutrients-12-01342-s001.zip › Supplementary Table 1.tiff]
